# Molecular mechanism of MLCK1 inducing 5-Fu resistance in colorectal cancer cells through activation of TNFR2/NF-κB pathway

**DOI:** 10.1007/s12672-024-01019-8

**Published:** 2024-05-12

**Authors:** Huifen Tang, Hui Zhou, Liang Zhang, Tingting Tang, Ning Li

**Affiliations:** Department of Hematology, The Affiliated Hospital, Hangzhou Normal University, 126# Wenzhou Road, Hangzhou, 310015 Zhejiang People’s Republic of China

**Keywords:** Myosin light chain kinase 1(MLCK1), Colorectal cancer (CRC), 5-Fluorouracil (5-Fu), Chemotherapy resistance, TNFR2/NF-κB pathway

## Abstract

**Background and aims:**

Chemotherapy resistance in colorectal cancer have been faced with significant challenges in recent years. Particular interest is directed to tumor microenvironment function. Recent work has, identified a small molecule named Divertin that prevents myosin light chain kinase 1(MLCK1) recruitment to the perijunctional actomyosin ring(PAMR), restores barrier function after tumor necrosis factor(TNF)-induced barrier loss and prevents disease progression in experimental inflammatory bowel disease. Studies have shown that MLCK is a potential target for affecting intestinal barrier function, as well as for tumor therapy. However, the relative contributions of MLCK expression and chemotherapy resistance in colorectal cancers have not been defined.

**Methods:**

Statistical analysis of MYLK gene expression differences in colorectal cancer patients and normal population and prognosis results from The Cancer Genome Atlas(TCGA) data. Cell activity was detected by Cell counting Kit-8. Cell proliferation was detected by monoclonal plate. The apoptosis was detected by flow cytometry and western blot. Determine the role of MLCK1 in inducing 5-Fluorouracil(5-Fu) resistance in colorectal cancer cells was detected by overexpression of MLCK1 and knock-down expression of MLCK1.

**Results:**

MLCK1 is expressed at different levels in different colorectal cancer cells, high MLCK1 expressing cell lines are less sensitive to 5-Fu, and low MLCK1 expressing cell lines are more sensitive to 5-Fu. MLCK1 high expression enhances resistance to 5-Fu in colorectal cancer cells and the sensitivity to 5-Fu was increased after knocking down the expression of MLCK1, that might be closely correlated to TNFR2/NF-κB pathway.

**Conclusions:**

MLCK1 high expression can enhance resistance to 5-Fu in colorectal cancer cells and the sensitivity to 5-Fu was increased after knocking down the expression of MLCK1, that might be closely correlated to TNFR2/NF-κB pathway, which will provide a new method for the treatment of colorectal cancer patients who are resistant to 5-Fu chemotherapy.

**Supplementary Information:**

The online version contains supplementary material available at 10.1007/s12672-024-01019-8.

## Introduction

Colorectal cancer (CRC) is a common malignancy of the digestive system and is one of the most common cancers in the world. According to GLOBOCAN data released by the National Cancer Institute, more than 1.88 million new colorectal cancer cases and 915,880 CRC-related deaths occurred in 2020, accounting for approximately one-tenth of cancer cases and deaths, the third highest incidence of all cancers (10.0%) and second in mortality (9.4%) of all cancers[1]. 5-Fu is a classical chemotherapeutic agent for colorectal cancer, which plays a key role in regulating of cell cycle and promoting apoptosis [2]. However, many patients have become unresponsive to 5-Fu during treatment, leading to recurrence and metastasis, therefore, chemotherapy resistance to 5-Fu has become an urgent issue.

**Fig.1:**
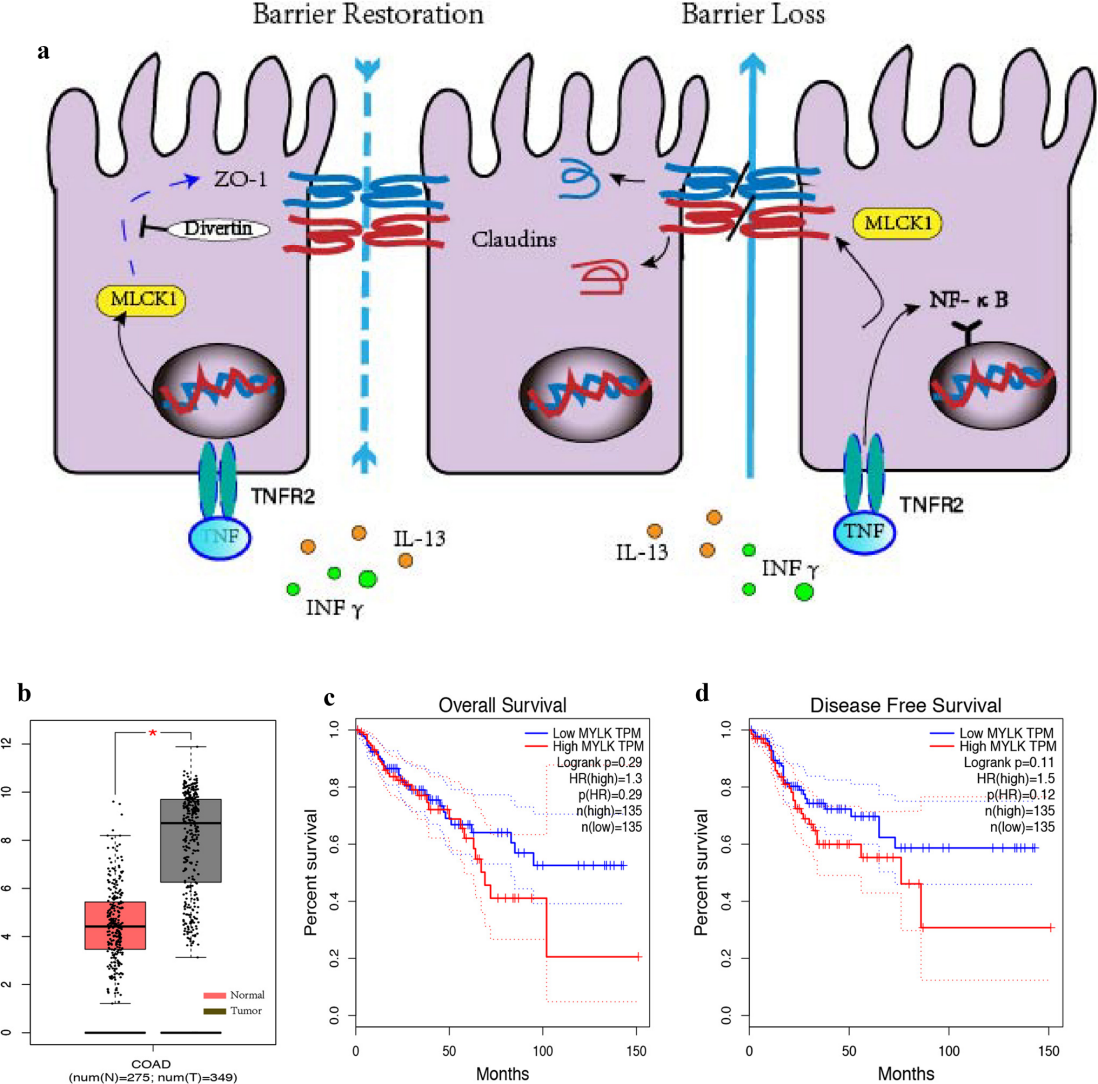
Mechanism of action of MLCK1 and analysis of data from TCGA.** a** recruitment of MLCK1 to the perijunctional actomyosin ring (PAMR) affects the binding of tight junction-related proteins such as ZO-1, claudins, and then increases the permeability of epithelial tight junctions, thereby causing a reduction in intestinal barrier function. A small molecule named Divertin that MLCK1 recruitment to the PAMR, restores barrier function and prevents disease progression in experimental inflammatory bowel disease caused by pro-inflammatory cytokine such as TNF, INF and et.al. **b** analysis of data from TCGA showed a significant difference in MLCK1 expression between normal and colorectal cancer patients (p < 0.01)**. c**, **d** no significant correlation with overall survival (OS) and disease-free survival (DFS)

On the one hand, chemotherapy resistance in colorectal cancer is related to epigenetic mutations, and intestinal structures such as mucosal barrier and microflora on the other hand. Particular interest is directed to tumor microenvironment function, which are the more promising processes that will be the subject of much research in the future [3]. Maintenance of intestinal mucosal homeostasis requires intestinal barrier function. Barrier dysfunction is thought to promote the development of intestinal and systemic diseases [4]. Expression of myosin light chain kinase 1(MLCK1) is closely associated with deficiency of intestinal mucosal barrier function [5, 6]. MLCK1 is encoded by the MYLK gene, which is a 210-kDa protein originally identified in cultured cells, embryonic tissues and endothelium [7–10], and the recruitment of MLCK1 to the perijunctional actomyosin ring (PAMR) affects the binding of tight junction-related proteins such as ZO-1, claudins, and then increases the permeability of epithelial tight junctions, thereby causing a reduction in intestinal barrier function (Fig. 1a). The recently discovered small molecule named divertin prevents the recruitment of MLCK1 to the PAMR, then reducing myosin light chain (MLC) phosphorylation and preventing the loss of intestinal barrier function [11]. MLCK1 expression plays an important role in the formation and progression of intestinal diseases by destroying the permeability of epithelial barrier [11, 12]. And it is associated with the production of pro-inflammatory cytokine in the inflamed intestinal tissues, such as TNF, INF and et al. [13, 14](Fig. 1a). Vitro models have been taken to show MLCK-mediated phosphorylation of myosin II regulatory light chain (MLC) was required for paracellular permeability increases that follow activation of Na + –nutrient cotransport [15], enteropathogenic E. coli infection [16], or TNF stimulation [17]. In addition, several recent reports have implicated the role of MLCK in animal models of inflammatory bowel disease (IBD) [12, 18, 19]. Moreover, the association between MLCK and colitis-associated cancer (CAC) has been reported that the specific role of NF-κB activation in the inflamed epithelia via TNFR2 signaling [12]. Finally, expression of MLCK increased para-cellular permeability in vitro [20, 21] and in vivo [22]. Therefore, MLCK is a key signaling node in physiological and pathophysiological regulation of epithelial tight junctions. Studies have shown that MLCK is a potential target for affecting intestinal barrier function, as well as for tumor therapy [11, 22]. However, the relative contributions of MLCK expression and chemotherapy resistance in colorectal cancers have not been defined and there’s very little report on this.

The involvement of the NF-κB pathway in tumor formation mechanisms, including tumor chemotherapy resistance and disease progression, has been widely reported in many researches [23]. An important mediator causing intestinal epithelial NF-κB activation is TNF-α, which is significantly elevated in the inflamed intestinal environment [24]. Specific upregulation of TNFR2 is observed in inflammatory intestinal epithelial cells and activates MLCK1-dependent dysregulation of tight junctions, leading to apoptosis-mediated barrier loss and experimental colitis [12] (Fig. 1a). The relationship between MLCK1 expression and TNFR2/NF-κB pathway is poorly understood.

Here we show the impaction and mechanism of MLCK1 in tumor therapeutic susceptibility, especially for chemotherapy resistance of colorectal tumor cells to 5-Fu. Analysis of data from TCGA showed a significant difference in MLCK1 expression between normal and colorectal cancer patients (p < 0.01) (Fig. 1b), but no significant correlation with overall survival (OS) (Fig. 1c) and disease-free survival (DFS) (Fig. 1d). Other than that, our experimental data may, in part, explain molecular mechanism of MLCK1 expression and TNFR2/NF-κB pathway in 5-Fu resistance of colorectal cancer cells, and these results need to be confirmed by additional experimental clinical studies.

## Materials and methods

### Cell lines and culture methods

The human colorectal cancer lines HCT116, RKO, SW480, LOVO, CACO2 and HT29 were purchased from Shanghai CAS Cell Repository. The cells were supplemented with 10% heat-inactivated fetal bovine serum at 37 °C with 5% CO2 and 95% humidity, and the culture medium was usually changed every 2 days. The fetal bovine serum was purchased from Corille (184590, Australia).

### Western blot assay

The proteins on the gel were transferred to the PVDF membrane, and the PVDF membrane was removed and closed in 10% skim milk closing solution; the membrane was washed with TBST and incubated with primary antibody at 4℃ overnight; the membrane was washed with TBST and incubated with secondary antibody at room temperature for 2 h; the membrane was washed with TBST, and the PVDF membrane was placed in a dark room and developed by dropwise addition of ECL color development working solution.

### Cell proliferation and viability assay

Cell viability was assessed using the Cell Counting Kit 8 (CCK-8) (LJ621, Dojindo, Japan) according to the manufacturer's instructions. Cells were inoculated at a density of 5,000 cells per well in 96-well flat-bottom microtitre plates. Incubate overnight by adding 100ul of medium containing various concentrations of 5-Fu and incubate for 24 h in an incubator. After incubation, aspirate the medium from the 96-well plate and add medium mixed with CCK8 (medium: CCK8 = 10:1) and continue incubate for 4 h at 37 °C. After 4 h, it’s time to stop incubation. The optical density values were determined in triplicate for the reagent blanks using a spectrophotometric enzyme marker at least at a reference wavelength of 450 nm. The experiment was repeated three times.

### Clone formation assays

High-expressing MLCK1 (CACO2, HT29) and low-expressing MLCK1 (LOVO, SW480) cells were inoculated in 6-cm dishes at a density of 2,000 cells per well, and then cultured and saturated with humidity in a cell culture chamber at 37 °C, 5% CO 2. When the clones could be seen under the microscope, the IC50 values of each cell line to 5-Fu were measured according to the previous CCK-8 method, and cultures containing different 5-Fu concentrations (control, 10ug/ml, 20ug/ml, and 100ug/ml) of the medium, changed the new medium every three days, and then continued the incubation for 2 weeks. The culture was terminated when visible macroscopic clones appeared. The supernatant was discarded and washed carefully twice with PBS. Fix with 4% formaldehyde for 15 min. Then remove the fixative, add appropriate amount of 0.25% crystal violet staining solution and stain for 15–20 min, then wash the stain slowly with running water and air dry. The plate was inverted and covered with a transparency film grid, and clones were counted directly with the naked eye, or more than 50 cells were counted with a microscope (low magnification). The experiment was repeated three times.

### Apoptosis assay

Flow cytometry was used to detect 5-Fu-induced apoptosis. Each procedure was performed strictly according to the instructions using the Annexin V-FITC / PI Apoptosis Assay Kit (556547) from BD, USA. The four screened cell lines were inoculated in 6 cm dishes at a density of 5 × 10 ^5 cells per well and then incubated in a cell incubator at 37 °C, 5% CO2 and saturated humidity. Incubate overnight, add different concentrations of 5-Fu medium (CACO2/LOVO group: control, 10ug/ml, 20ug/ml, 100ug/ml; HT29/SW480 group: control, 20ug/ml, 40ug/ml, 200ug/ml) for 24 h. Collect all cell cultures and cells, centrifuge at 800 g for 5 min and remove supernatant. Cells were washed with cold PBS, centrifuged, supernatant discarded, and then resuspended by adding 1 ml of 1 × binding buffer and adjusting the cell concentration to 1*10 ^6 cells/ml. 100ul (10 ^5 cells) of cell suspension was added to the flow tubes, and 5ul of FITC-Annexin V and 5ul of PI were added to each flow tube. The cells were mixed with the stain and then left in the dark at room temperature for 15 min. Then 400ul of 1 × binding buffer was added to each flow tube and tested on the machine. Annexin V-FITC showed green fluorescence and PI showed red fluorescence. The experiment was repeated three times.

### Plasmid transfection

Lipofectamine TM 2000 Transfection Reagent (11668019) was used to transfect the MLCK1 low expression cell lines LOVO and SW480. Transfection was performed according to the manufacturer's instructions (MiaoLing Plasmid Platform, China). The LOVO and SW480 cells were inoculated in 6 cm dishes at a density of 5 × 10^5 cells per well. Incubate overnight to reach 70–80% cell fusion. Add 50ul of OPTI-MEM to two 1.5 ml EP tubes, add 3ug of plasmid to one tube, add 9μl of Lipofectamine 2000 to one tube, and then add OPTI-MEM containing Lipofectamine 2000 to OPTI-MEM with plasmid. After mixing, it was left at room temperature for 5 min, then it was added dropwise to a Petri dish and gently shaken, mixed and incubated in an incubator for 6 h, and then switched to complete medium and continued to incubate.

### Statistical analysis

The data obtained from the experiment were expressed as W mean ± standard deviation (S ± s), and one-way ANOVA was performed using SPSS.17 statistical software, and the qualitative response data were tested by X2 test, and P < 0.05 was considered statistically significant difference.

## Results

### MLCK1 is expressed at different levels in different colorectal cancer cells, high MLCK1 expressing cell lines are less sensitive to 5-Fu, and low MLCK1 expressing cell lines are more sensitive to 5-Fu

**Fig.2:**
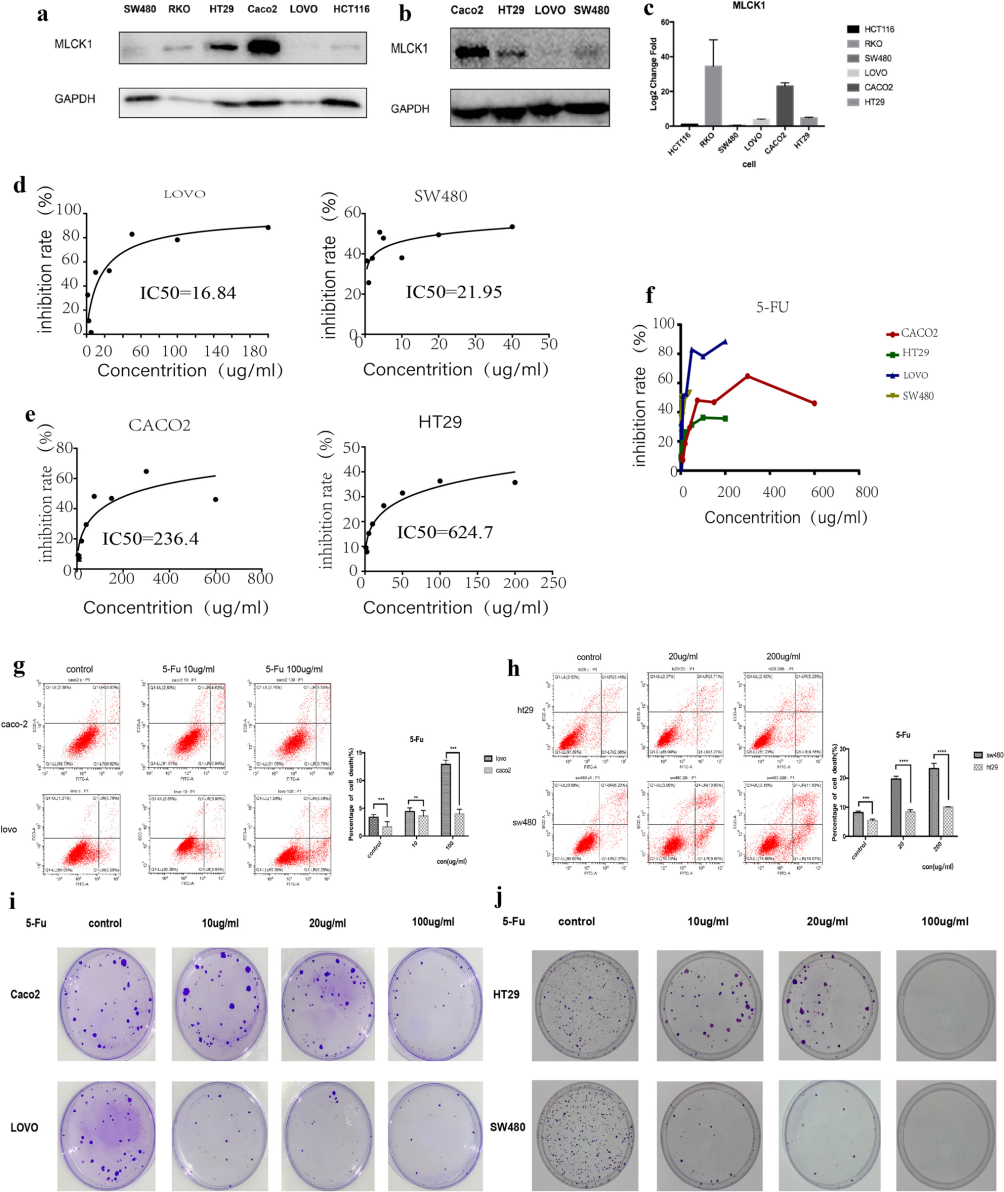
MLCK1 was expressed at different levels in different colon cancer cells, and high expression MLCK1 cell lines were less sensitive to 5-Fu.** a**–**c** results of western blot and RT-PCR show that expression levels of MLCK1 were higher in CACO2 and HT29 cells and lower in LOVO and SW480. **d**, **e** 24h IC 50 of 5-Fu for LOVO cells was 16.84ug/ml, for SW480 cells was 21.95ug/ml, which are much lower than that of HT29 cells(624.7ug/ml) and CACO2 cells(236.4ug/ml)**. f** results of inhibition rate suggest that at a certain low concentration range, 5-Fu inhibited HT29 cells and CACO2 cells much more than SW480 cells and LOVO cells**. g**, **h** we divided the cells into two groups (CACO2 vs LOVO and HT29 vs SW480), and treated them with different concentrations of 5-Fu (10ug/ml, 100ug/ml vs control and 20ug/ml, 200ug/ml vs control), the flow apoptosis results suggested that HT29 and CACO2 cells have little apoptosis in the 5-Fu group which were not sensitive to 5-Fu. More cells apoptosis in SW480 and LOVO group, and apoptosis was significantly higher, statistically significant difference (p < 0.05)**. i**, **j** flat dish monoclonal experiment, divided into two groups, with different concentrations of 5-Fu(control/10/20/100ug/ml), and the results showed that at the same concentration of 5-Fu, SW480 cells and LOVO cells had fewer clones than HT29 cells and CACO2 cells

In our experiment, we extracted RNA and protein from 6 colorectal cancer cell lines and confirmed the different expression levels of MLCK1 in different colorectal cancer cells by western blot and RT-PCR, and results suggested that the expression levels of MLCK1 were higher in CACO2 and HT29 cells and lower in LOVO and SW480(Fig. 2a–c). As seen in Fig. 2d, the 24h IC 50 of 5-Fu for LOVO cells was 16.84ug/ml, for sw480 cells was 21.95ug/ml, which seem to be much lower than that of HT29 cells(624.7ug/ml) and CACO2 cells(236.4ug/ml) (Fig. 2e). In addition, the 4 cell lines were treated with various concentrations of 5-Fu, the results of inhibition rate suggest that at a certain low concentration range, 5-Fu inhibited HT29 cells and CACO2 cells much more than SW480 cells and LOVO cells. However, when the 5-Fu concentration reached a certain high value, the above results were not taken as above (Fig. 2f). To further investigate that low-expressing MLCK1 cells are more sensitive to 5-FU than high-expressing MLCK1 cells, we used flow cytometry to detect the apoptosis rates of cells (Fig. 2g, h). After we divided the cells into two groups (CACO2 vs LOVO and HT29 vs SW480), and treated them with different concentrations of 5-Fu (10ug/ml, 100ug/ml vs control and 20ug/ml, 200ug/ml vs control), the flow apoptosis results suggested that HT29 and CACO2 cells have little apoptosis in the 5-Fu group which were not sensitive to 5-Fu. More cells apoptosis in sw480 and LOVO group, and apoptosis was significantly higher, statistically significant difference (p < 0.05). Moreover, we performed a flat dish monoclonal experiment, divided into two groups, with different concentrations of 5-Fu, and the results showed that at the same concentration of 5-Fu, SW480 cells and LOVO cells had fewer clones than HT29 cells and CACO2 cells (Fig. 2i, j). The 24-h IC50 values measured by cell proliferation and viability assay, flow apoptosis assay, and proliferation and clone formation assay suggested that the cell lines with high MLCK1 expression (CACO2 and HT29) was less sensitive to 5-Fu than that of the cell lines with low MLCK1 expression (LOVO and SW480).

Given these results, we further studied the mechanism of action of MLCK in intestinal epithelial cells and we focused here on the long MLCK, MLCK1. Three distinct MLCK genes are present in mammals [25, 26]. Here, we need to better understand the distinct functional roles of the long MLCK (220-kDa, MLCK1 and 130-kDa, MLCK2) forms of myosin light chain kinase. Only isoforms MLCK1 and MLCK2, which differ by a single 207 nucleotide exon, are expressed in intestinal epithelia [10, 27, 28]. In the human small intestine, MLCK1 expression is restricted to villous enterocytes, where it is concentrated at the PAMR which has been mentioned previously and caused chronic immune-mediated disease [11]. In professor W. Vallen Graham’s study [29], they report an alternative strategy for the therapeutic inhibition of long MLCK-dependent barrier loss, and they show that MLCK1, but not MLCK2, is recruited to the PAMR and caused barrier dysfunction. Further study demonstrated that, beyond MLCK enzymatic activity, transcriptional upregulation of MLCK1 expression was essential to TNF-induced barrier loss [14, 30]. TNF activated MLCK1 transcription via the high-affinity TNF receptor TNFR2, thereby explaining TNF were sufficient to trigger MLCK upregulation and barrier dysfunction in vitro at extremely low concentrations [30]. Consistent with this, in vivo studies demonstrated that TNFR2 signaling was required for epithelial MLCK1 upregulation during disease progression in T cell transfer-induced, experimental, chronic inflammatory bowel disease [12, 31].

### MLCK1 high expression enhances resistance to 5-Fu in colorectal cancer cells and the sensitivity to 5-Fu was increased after knocking down the expression of MLCK1, that might be closely correlated to TNFR2/NF-κB pathway

**Fig.3:**
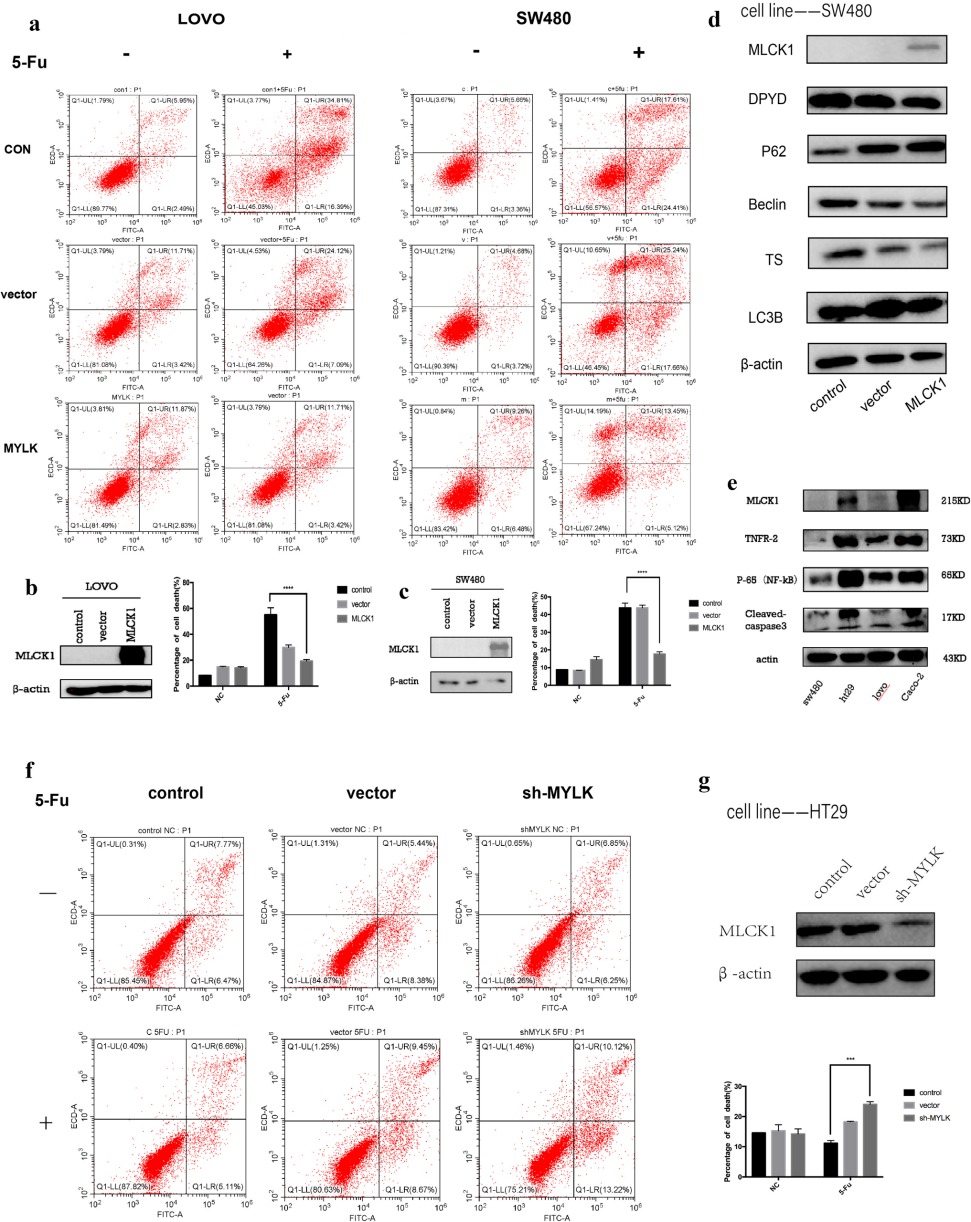
Transient overexpression of MLCK1 and knockdown expression of MLCK1.** a**–**c** after plasmid transfection of two screened low-expressing MLCK1 cell lines (LOVO and SW480) overexpressing MLCK1, and treated them with different concentrations of 5-Fu (200ug/ml in LOVO cell line and 400ug/ml in SW480 cell line) for 24h, cell death in control and dosing groups was detected using flow apoptosis, and the results suggested that for LOVO and SW480 cell lines, colon cancer cells were significantly less sensitive and more resistant to 5-Fu after overexpressing MLCK1, the p < 0.05, statistically significant difference. **f**, **g** the sensitivity of HT29 colon cancer cells to 5-Fu was significantly increased after knocking down the expression of MLCK1(sh-MYLK), and the flow cytometry results after 5-Fu adding suggested higher cell mortality, p < 0.05, statistically significant difference. **d** for SW480 cell line, the expression levels of autophagy-related proteins, i.e., LC3B II and Beclin-1 decreased and P62 increased after plasmid transfection with high expression of MLCK1, suggesting a decrease in death autophagy levels. **e** results of western blot after protein extraction of the four cell lines selected in the primary screen suggested that TNFR2 and NF-κB (P65) protein expression was higher in cell lines with higher expression of MLCK1 (HT29, CACO2) and lower in cell lines with lower expression of MLCK1 (LOVO and SW480)

After plasmid transfection of two screened low-expressing MLCK1 cell lines (LOVO and SW480) overexpressing MLCK1, cell death in control and dosing groups was detected using flow apoptosis, and the results suggested that for LOVO and SW480 cell lines, colon cancer cells were significantly less sensitive and more resistant to 5-Fu after overexpressing MLCK1 (Fig. 3a), the p < 0.05, statistically significant difference (Fig. 3b, c). For the cell line HT29 with high expression of MLCK1, the sensitivity of HT29 colon cancer cells to 5-Fu was significantly increased after knocking down the expression of MLCK1, and the flow cytology results after 5-Fu adding suggested higher cell mortality, p < 0.05, statistically significant difference (Fig. 3f, g).

The results of western blot after protein extraction of the four cell lines selected in the primary screen suggested that TNFR2 and NF-κB (P65) protein expression was higher in cell lines with higher expression of MLCK1 (HT29, CACO2) and lower in cell lines with lower expression of MLCK1 (LOVO and SW480) (Fig. 3e), which suggests that MLCK1 expression is inextricably linked to the TNFR2/NF-κB pathway. This has been illustrated in the subsequent cell sequencing, where we collated data related to the TNFR2/NF-κB pathway. Suzuki M professor previously observed that the NF-κB activation in colonic epithelial cells is associated with increased TNFR2 expression in CAC development[32]. As we know, detection of cleaved-caspase3 is important for the detection of apoptosis, and expression of cleaved-caspase3 confirms apoptosis (Fig. 3e). From the western-blot results, the higher expression of MLCK1 proteins (HT29 and CACO2) have an increased caspase3 cleavage. This further suggests that high-expressing MLCK1 cells are more potent in TNFR2/NF-κB pathway than cleaved-caspase3-mediated apoptosis, thereby protecting cells from death. Of course, these need to be confirmed by further research.

In addition, the results of western blot of cellular proteins before and after transfection suggested that there were changes in the expression levels of autophagy-related proteins, i.e., LC3B II and Beclin-1 decreased and P62 increased after plasmid transfection with high expression of MLCK1, suggesting a decrease in autophagy levels (Fig. 3d), and consulting that the resistance of MLCK1 high expression cells to chemotherapy was associated with a decrease in cellular autophagy levels. As seen in Fig. 3d, western blot revealed there’s changes in the expression of dihydropyrimidine dehydrogenase (DPYD) and thymidylate synthetase (TS), reduced expression of DPYD after overexpression of MLCK1. DPYD is responsible for the degradation of 5-fluorouracil (5-FU) [33, 34]. The DPYD gene encodes dihydropyrimidine dehydrogenase (DPD), an enzyme that catalyzes the rate-limiting step in fluorouracil metabolism. High DPD activity in tumor resulted in poor prognosis, especially in patients who received 5-FU-based adjuvant chemotherapy and that is associated with hyposensitive of 5-Fu [35, 36]. Instead, we obtained the opposite result, i.e., decreased expression of DPYD after overexpression of MLCK1, and the TS was the same. Given these results, we suggest that MLCK1 overexpression further affects 5-FU metabolism through other important pathways to induce drug resistance in colorectal cancer cells.

### Overexpression of MLCK1 by stable transfection of colorectal cancer cells showed enhanced resistance to 5-Fu, consistent with the transient transfection results, and the sequencing results further suggested that this was associated with the TNFR2/NF-κB pathway

**Fig.4:**
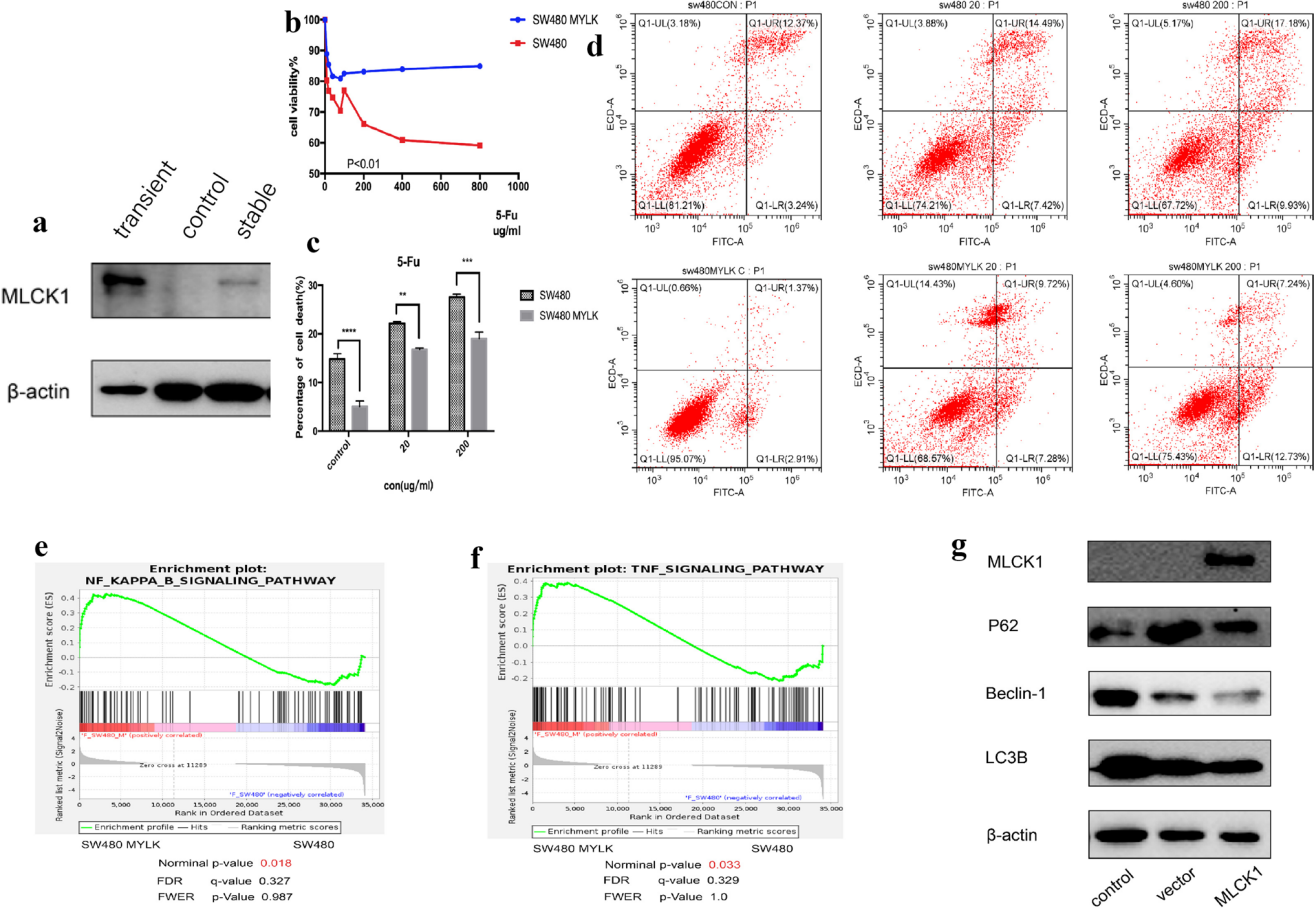
Results of stable transcendental overexpression of MLCK1 in SW480 cell.** a** result of western-blot of control/transient/stable transcendental overexpression of MLCK1 in SW480 cell line.** b** results of cell inhibition rate at different 5-Fu concentrations suggested the drug inhibited SW480 MYLK cell line at a much lower rate than SW480 cell line**. c**, **d** at different concentrations of 5-Fu administration (20ug/ml; 200ug/ml) with the control group suggest that after stable transfection of overexpressed MLCK1 apoptosis of SW480 cells was reduced, p < 0.05, statistically significant difference. **e**, **f** we performed cell sequencing of the stably transfected MLCK1 overexpressing cell line SW480 MYLK, and the results suggested that the enhanced drug resistance may be achieved by MLCK1 through TNFR2/NF-κB pathway interactions**. g** autophagy-related pathway proteins changed after stable transfection with MLCK1 overexpression in SW480 MYLK, i.e., LC3B II/Beclin-1 expression decreased and P62 expression increased, indicating that the level of death autophagy in intestinal cancer cells decreased

**Fig. 5:**
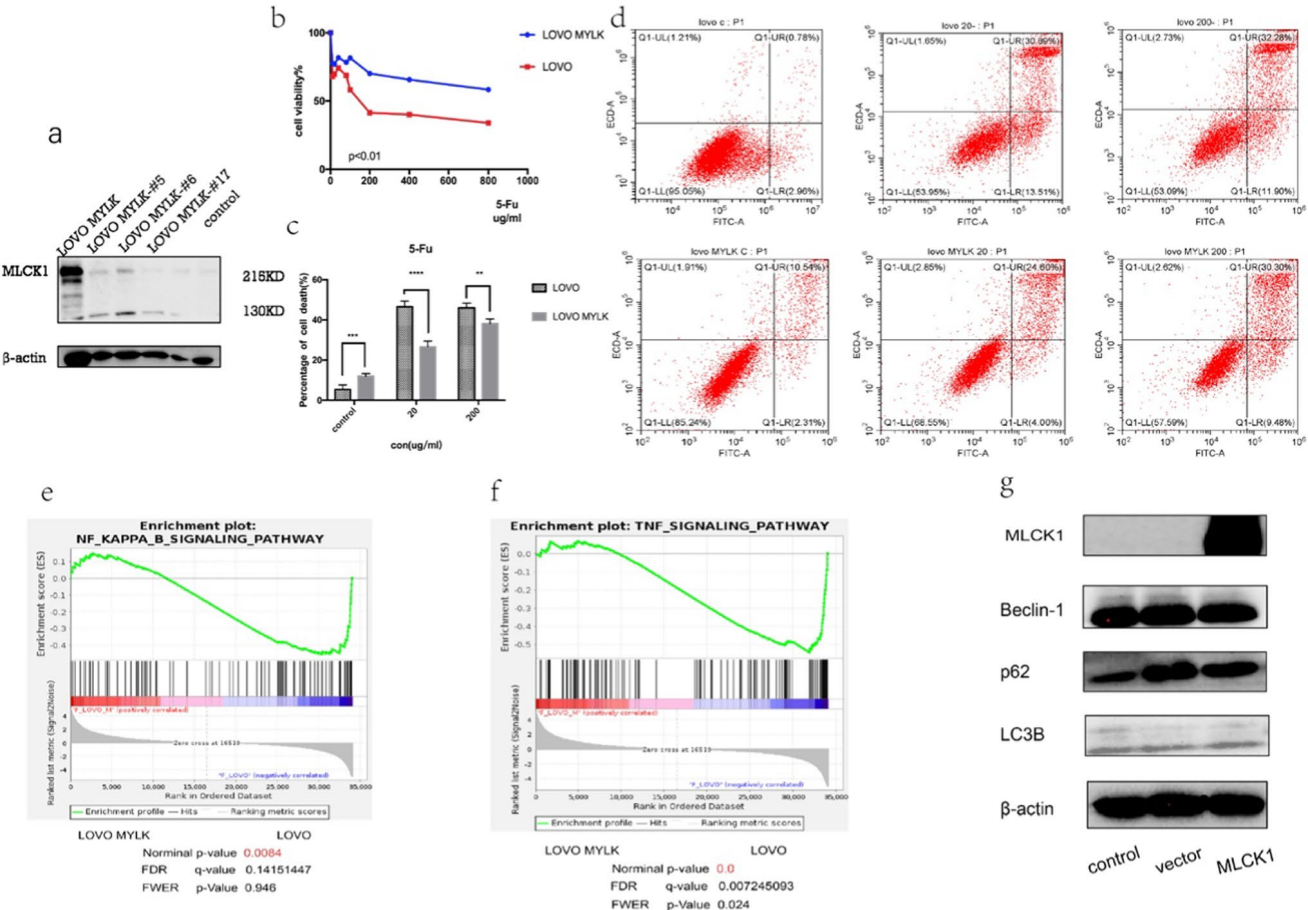
Results of stable transcendental overexpression of MLCK1 in SW480 cell.** a** result of western-blot of control/transient/stable transcendental overexpression of MLCK1 in LOVO cell line (#5#6#17 means different stable transcendental clone numbers)**. b** results of cell inhibition rate at different 5-Fu concentrations suggested the drug inhibited LOVO MYLK cell line at a much lower rate than LOVO cell line**. c**, **d** at different concentrations of 5-Fu administration (20ug/ml; 200ug/ml) with the control group suggest that after stable transfection of overexpressed MLCK1 apoptosis of LOVO cells was reduced, p < 0.05, statistically significant difference**. e**, **f** we performed cell sequencing of the stably transfected MLCK1 overexpressing cell line LOVO MYLK, and the results are consistent with SW480 MYLK cell line**. g** after stable transfection with MLCK1 overexpression in LOVO MYLK, i.e., LC3B II/Beclin-1 expression decreased and P62 expression increased, indicating that the level of autophagy in intestinal cancer cells decreased

As seen the Fig. 4a, after SW480 cell lines stably transfected with MLCK1 overexpressing, cell death in control and dosing groups was detected using flow apoptosis. The results at different concentrations of 5-Fu administration (20ug/ml; 200ug/ml) with the control group suggest that after stable transfection of overexpressed MLCK1 apoptosis of SW480 cells was reduced(Fig. 4d), p < 0.05(Fig. 4c). Additionally, the results of cell inhibition rate at different 5-Fu concentrations, which suggested the drug inhibited SW480 MYLK cell line at a much lower rate than SW480 cell line in Fig. 4b. When we similarly stably transfected overexpressing MLCK1 in the LOVO cell line, consistent results came out to suggest that stably transfected overexpressing cells were less sensitive to 5-Fu than ordinary cells (Fig. 5a–d). Subsequently, we performed cell sequencing of the stably transfected MLCK1 overexpressing cell lines SW480 MYLK and LOVO MYLK, and the results suggested that the enhanced drug resistance may be achieved by MLCK1 through TNFR2/NF-κB pathway interactions(Figs. 4e, f, 5e, f). In addition, after performing western-blot on protein extracted from stably transfected cells overexpressing MLCK1. We found that autophagy-related pathway proteins changed after stable transfection with MLCK1 overexpression, i.e., LC3B II//Beclin-1 expression decreased and P62 expression increased, indicating that the level of death autophagy in intestinal cancer cells decreased (Figs. 4g, 5g). However, our conclusion need to be confirmed by more experimental results.

Given these results, considering the mechanism of action of MLCK1 in intestinal epithelial cells, we hypothesized that the expression of MLCK1 is a significant factor for the mechanism of drug resistance in colorectal cancer cells. That may be closely associated with activation of the TNFR2/NF-κB pathway. In addition, a possible decrease in autophagy levels may also get involved. As we know, MLCK1 plays an important role in many intestinal disorders such as IBD and CAC, and both knockout of MLCK and treatment of experimental mice with highly specific inhibitors cause deficits in intestinal barrier function [31]. Therefore, we made the hypothesis that the loss of intestinal barrier function also has an effect on the absorption effect of drugs. More experiments are needed to demonstrate this in the next in vivo experiments in animals.

The involvement of MLCK1 in colonic mucosal homeostasis and the progression of colonic disease has been extensively studied by W. Vallen Graham et al. [11, 29]. They found that myosin light chain kinase (MLCK) increased by TNF both in vitro and in vivo is a key effector and potential therapeutic target for barrier dysfunction, even though the special inhibition has some side effects. Moreover, they have reported that MLCK promoter activity is mediated by NF-κB activation consistent with the findings of previous groups [37, 38]. Other than this, Suzuki M et al. suggested that disrupted epithelial tight junction (TJ) and elevated pro-tumorigenic cytokines may be associated with CAC development, which are essentially induced by MLCK expression and TNFR2 signaling pathway [32]. In this regard, it has been reported by Wang et al. that the up-regulated MLCK in human intestinal cells is required for TNF-induced collapse of epithelial TJ by TNFR2 signaling[14, 30]. These results imply that the expression of MLCK produced by TNFR2/NF-κB may be associated with the development of CAC and the chemotherapy resistance in colorectal cancers.

## Discussion

Epithelial barrier loss is a critical component of acute and chronic gastrointestinal diseases, such as food allergy, celiac disease, infectious enterocolitis, IBD and even neoplasms [39]. At advanced stages, colitis progresses might result in malignant colorectal disease including colitis-associated carcinoma (CAC), through proven pathways such as autophagy and mucosal barrier disruption [40]. Importantly, intestinal inflammation caused by disruption of the intestinal mucosal barrier and bacterial invasion, which bring about inflammatory cytokine like NF-κB, TNF-α, and IL-6, inflammatory cell infiltration, all of these contribute to survival advantages to abnormal proliferation in cancer cells [41]. However, signaling pathways such as TNF, NF-κB and so on are stimulated by exposure of the tumor to radiation or chemotherapy drugs in the tumor microenvironment (TME), leading to resistance of cancer cells to apoptosis, as well as promoting angiogenesis and tumor growth. These may cause chemotherapy resistance [42, 43]. Our data suggest that preservation of the epithelial barrier may be beneficial in preventing carcinoma development and chemotherapy resistance.

Consistently, We show here that MLCK1 high expression can enhance resistance to 5-Fu in colorectal cancer cells and the sensitivity to 5-Fu was increased after knocking down the expression of MLCK1, that might be closely correlated to TNFR2/NF-κB pathway. Briefly, MLCK1 knockout might prevent chemotherapy resistance by recovering epithelial barrier loss and altering the tumor microenvironment(TME) through TNFR2/NF-κB pathway. However, we need more experimental studies to confirm this hypothesis. So far, researches have shown that long MLCK knockout in vivo, which has no apparent toxicity, is beneficial in immune-mediated colitis [12]. Others reported that enzymatic MLCK inhibition could not be used therapeutically because MLCK knockout leads to hypotension, bladder dysfunction, severe intestinal dysmotility, and death [44]. this This limitation overcame by development of inducible knockout mice with loxP sites flanking the MYLK sequence encoding the catalytic domain [45]. Thus, while long MLCK inhibition maybe useful in oncology treatment, loss of MLCK enzymatic activity in smooth muscle [45] and non-muscle [12, 46] cells would have unacceptable toxicities.

MLCK is a key signaling node in physiological and pathophysiological of epithelial tight junctions by regulating Tight Junction (TJ) protein Interactions and structure. Previous research reported that epithelial NF-κB activation plays a role in opening paracellular spaces through dysfunction of tight junction proteins such as occludin, claudin-1 and zona occludens-1 [47]. An increase in MLCK protein expression in vitro and in vivo mediated TNF-induced barrier loss [31, 48]. However, in vitro studies from two groups conflict as to whether TNF-induced MLCK upregulation depended on NF-κB or AP-1 signaling [29, 30, 37]. A group found that the NF-κB inhibitors such as curcumin prevented TNF-induced MLCK upregulation and barrier loss [38, 49]. But another group found that a series of NF-κB inhibitors, such as curcumin, MG132, capsaicin and triptolide were unable to prevent TNF-induced barrier loss [14]. Interestingly, this group found that sulfasalazine was able to block TNF-induced barrier loss in a dose dependent manner which prevent barrier loss at low dose but exaggerate barrier loss at high dose [14].

The human long MLCK promoter has been cloned by different research groups, which contained functional binding sites for both NF-κB and AP-1 [29, 37]. In the common model, undifferentiated intestinal epithelial cells modestly upregulate long MLCK via NF-κB signaling, while differentiated intestinal epithelial cells upregulate long MLCK induced by TNF via AP-1 signaling. Additionally, one group has shown that AP-1-activating elements such as MAP kinases, are involved in TNF-induced long MLCK upregulation [50, 51]. These data might separate NF-κB from MLCK upregulation and suggest that another signaling pathway activated by TNF was responsible for barrier loss. At this point, the relationship between MLCK, NF-κB and TNF is becoming more clear which also provides a basis for the reversal of MLCK-related barrier loss by pharmacotherapy such as TNF inhibitor, NF-κB inhibitor, specific MLCK1 inhibitor and other inhibitors of inflammatory factors.

More interestingly, recent observation reported that an enzymatically inactive region, IgCAM3 [52], was responsible for long MLCK1 recruitment to the PAMR, blocking MLCK-dependent tight junction regulation in intestinal disease. They found a molecule, Divertin, was able to displace long MLCK1 from the PAMR, reverse TNF-induced barrier loss in vivo and in vitro [11]. Thus, divertin might be useful to reverse and prevent acute TNF-induced MLC phosphorylation and barrier loss in inflammatory diseases and play important role in treatment of malignant of the intestine. Conclusively, despite the difficulties in the treatment of intestinal malignancies have been overcome in the past few years, there remains much to be learned. Specific topics such as elucidation of mechanisms that regulate MLCK1 in loss of epithelial barrier function and further definition of pore pathway function in health and disease, and characterization of the structural and functional properties of defined and to-be-discovered in molecular mechanism of MLCK1 in preventing colorectal cancer chemotherapy resistance, which need more time and practice to confirm.

### Supplementary Information

Supplementary Material 1.

## Data Availability

All data generated or analyzed during this study are included in this published article.

## Abbreviations

MLCK1: Myosin light chain kinase 1
PAMR: Perijunctional actomyosin ring
TNF: Tumor necrosis factor
TCGA: The Cancer Genome Atlas
5-Fu: 5-Fluorouracil
CRC: Colorectal Cancer
MLC: Myosin light chain
IBD: Inflammatory bowel disease
CAC: Colitis-associated cancer
OS: Overall survival
DFS: Disease-free survival
CCK-8: Cell Counting Kit 8
DPYD: Dihydropyrimidine dehydrogenase
TS: Thymidylate synthetase
DPD: Dihydropyrimidine dehydrogenase
TJ: Tight junction
TME: Tumor microenvironment
MAP: Mitogen activated protein
