# Supplementary material for: Molecular mechanism of MLCK1 inducing 5-Fu resistance in colorectal cancer cells through activation of TNFR2/NF-κB pathway
Source: Discov Oncol. 2024 May 12;15:159. doi: 10.1007/s12672-024-01019-8 (PMC11089027; doi:10.1007/s12672-024-01019-8)
Supplement: Supplementary file 1 — Supplementary Material 1. [file 12672_2024_1019_MOESM1_ESM.pdf]

Figure2

Tips：Some of the clearer original images may be confusingly named, but we try to provide the full original image wherever possible. Too much time has been spent in the preliminary peer review, and the current submission is urgent, so we hope it will be passed quickly, thank you!

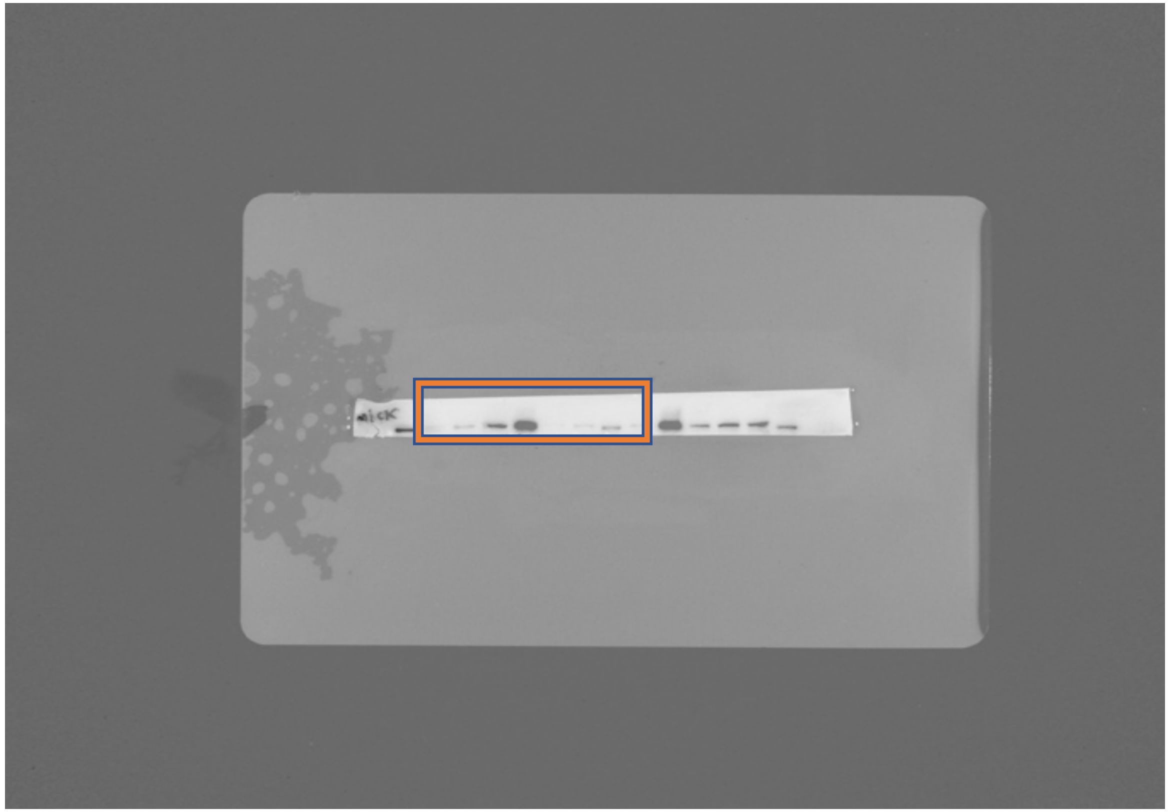

MLCK1

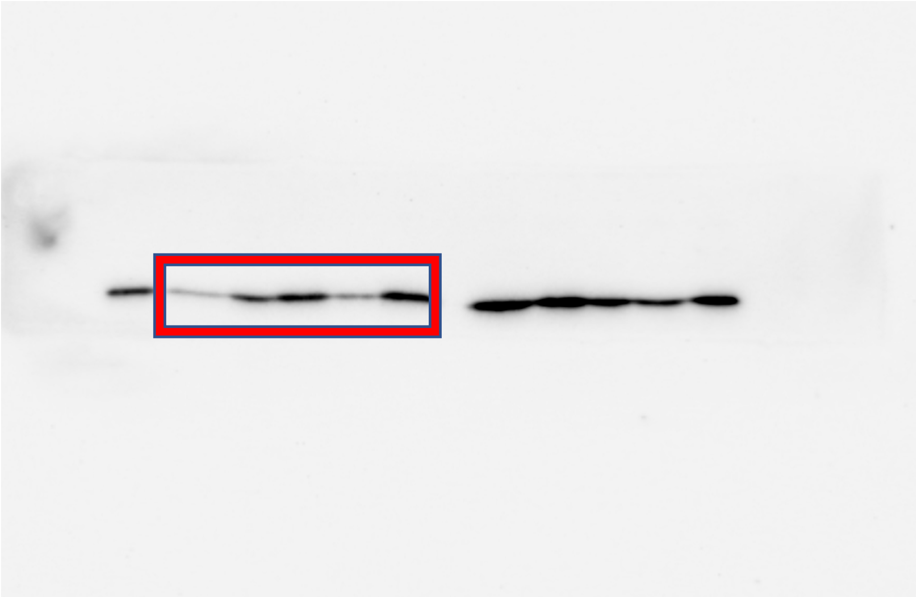

GAPDH

Figure2

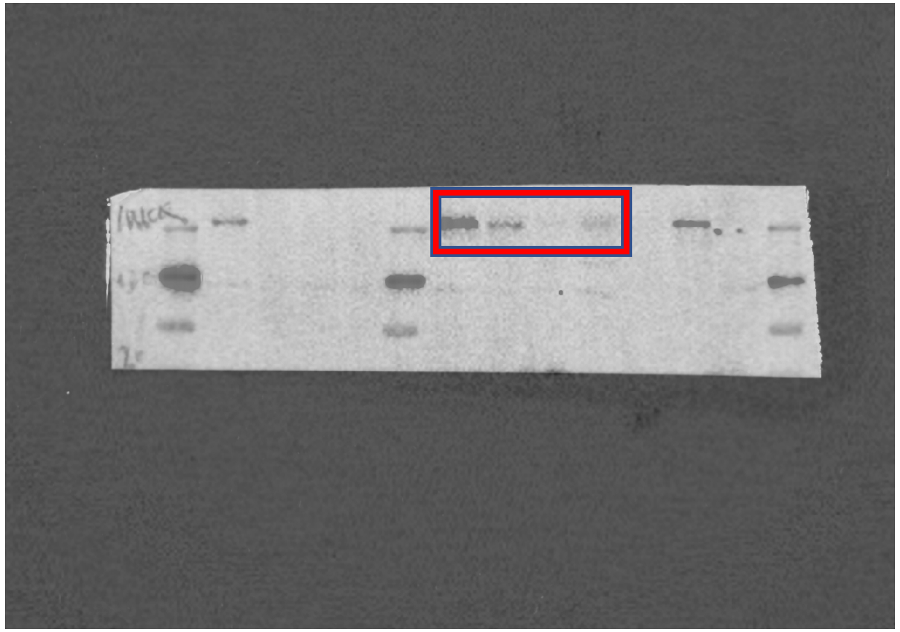

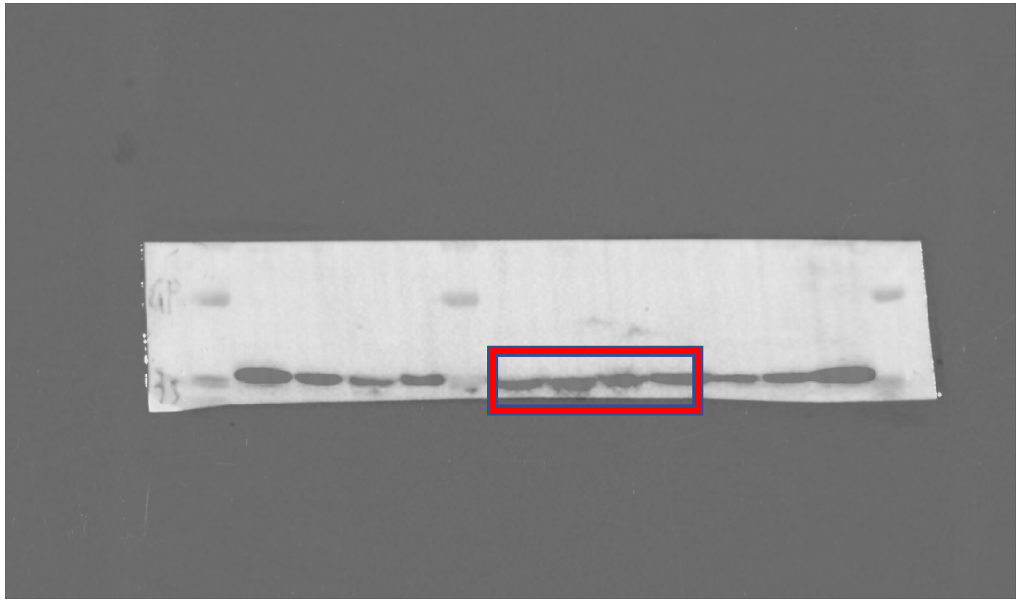

MLCK1

GAPDH

Figure3

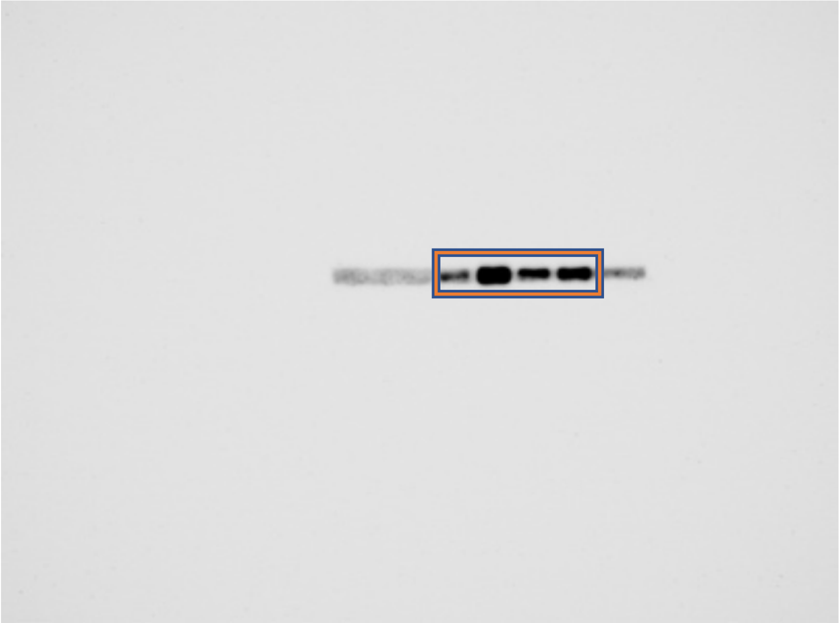

P65/NF-kB

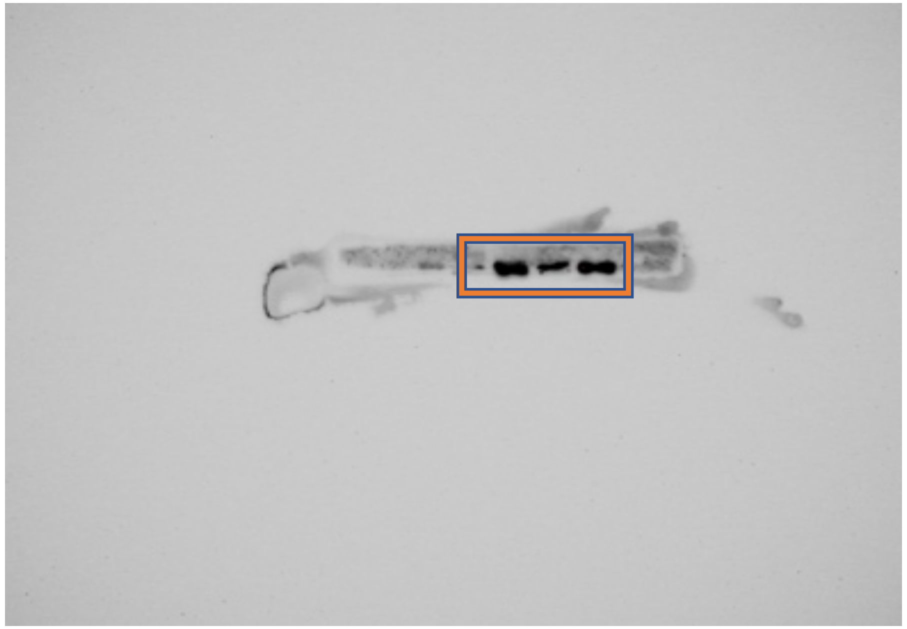

TNFR2

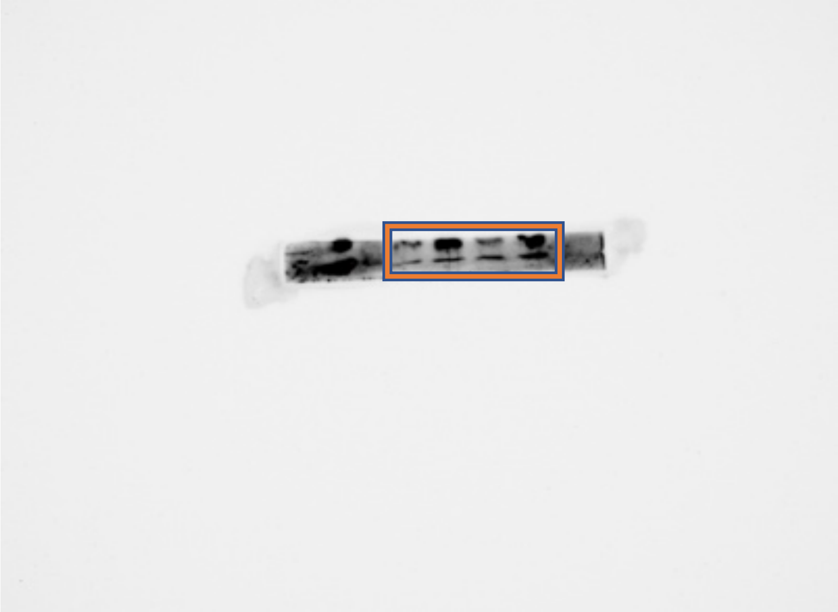

Cleaved-casepase3

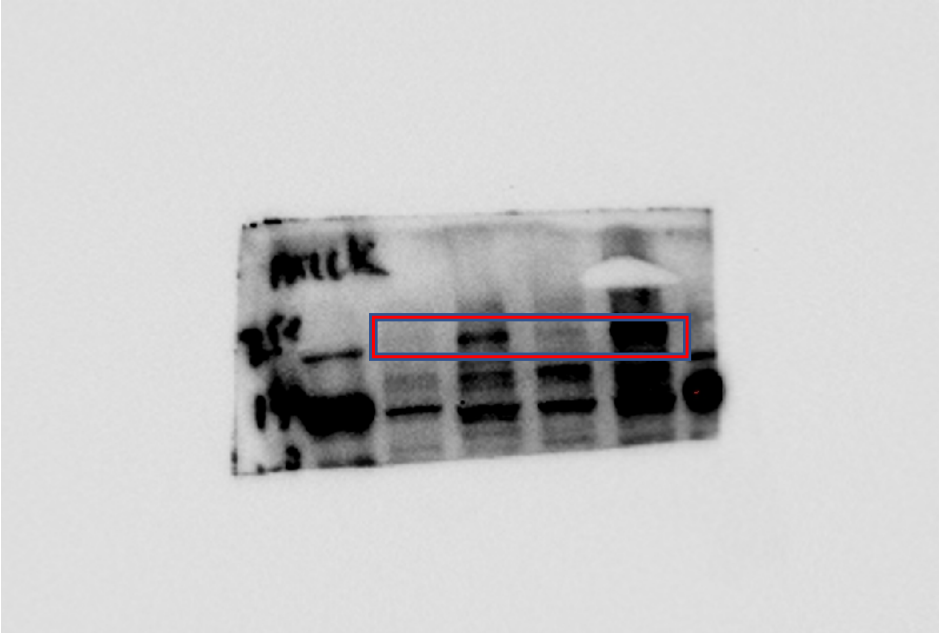

MLCK1

Figure3

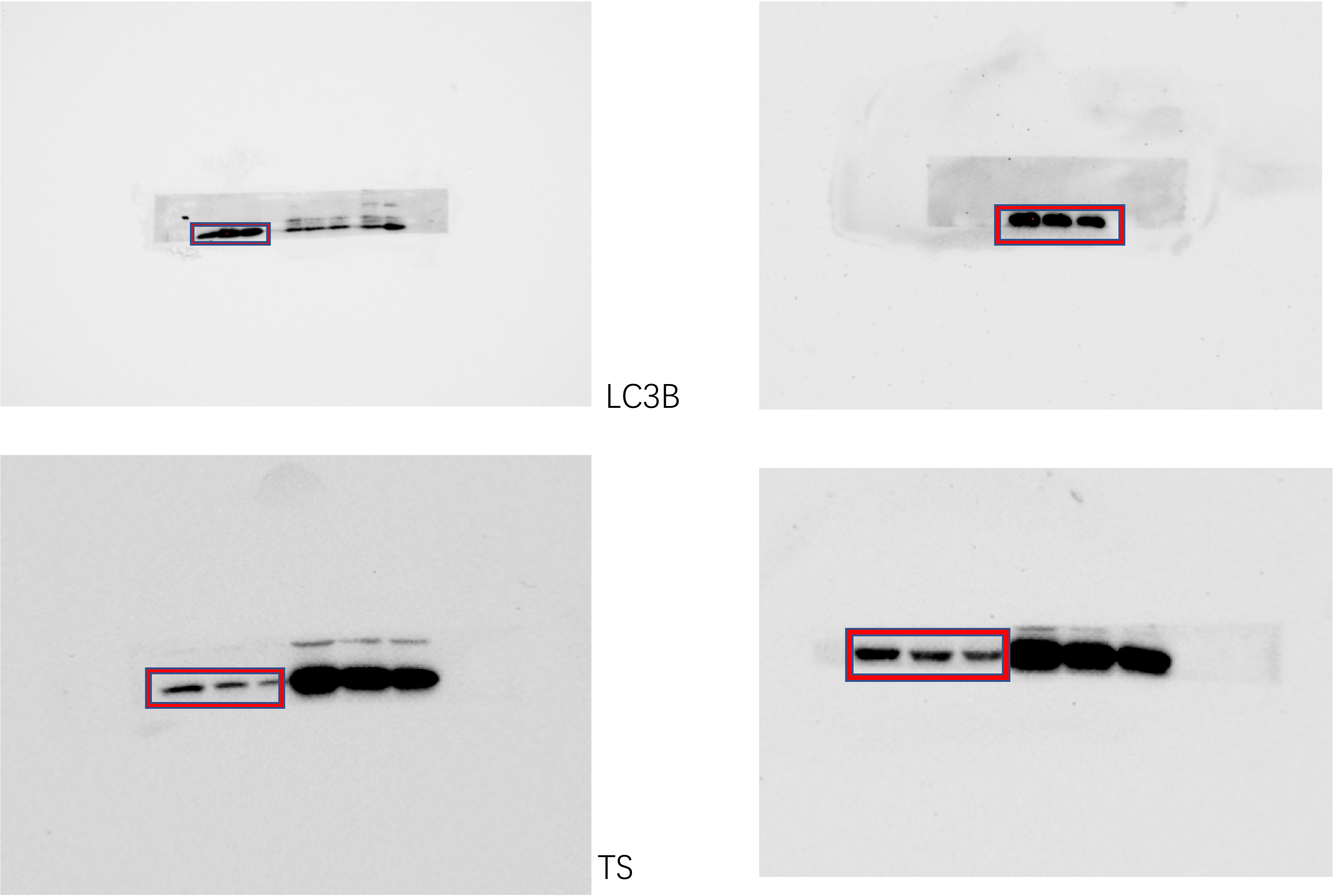

LC3B

DPYD

TS

Beclin

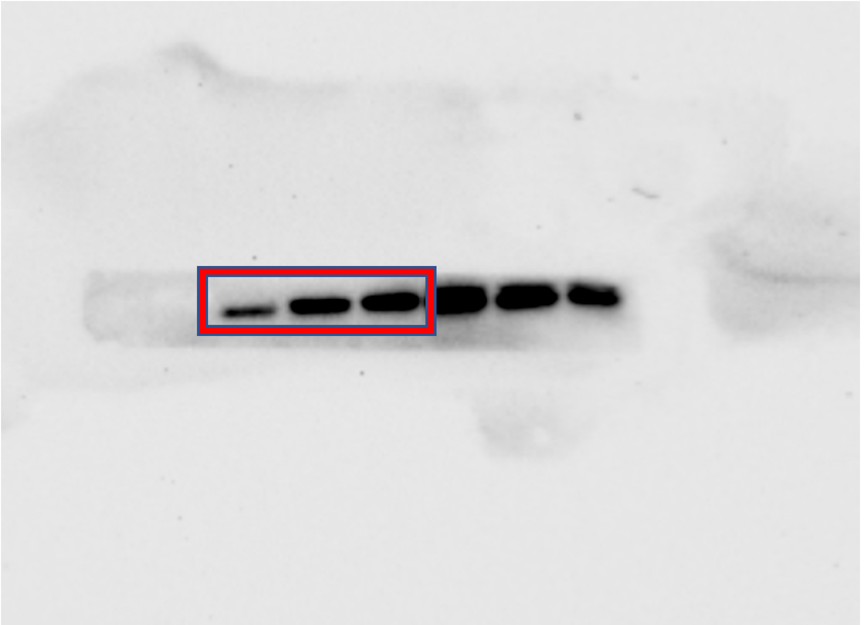

P62

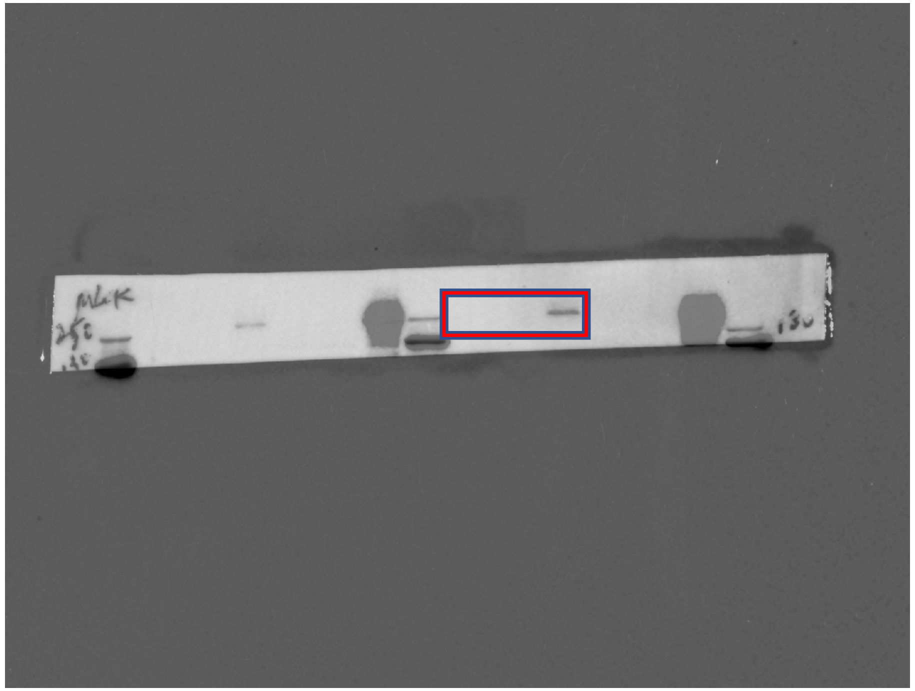

MLCK1

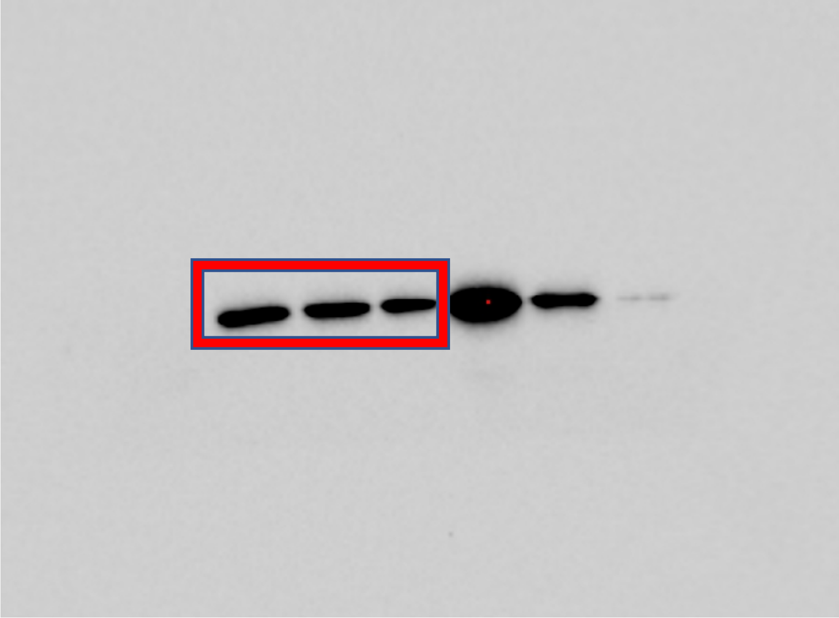

β-actin

Figure4

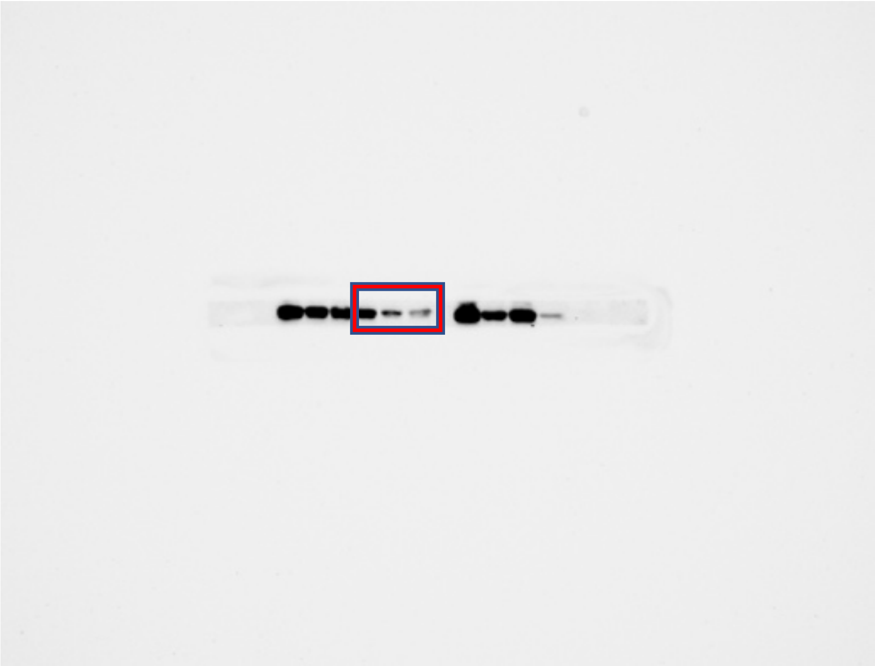

Beclin

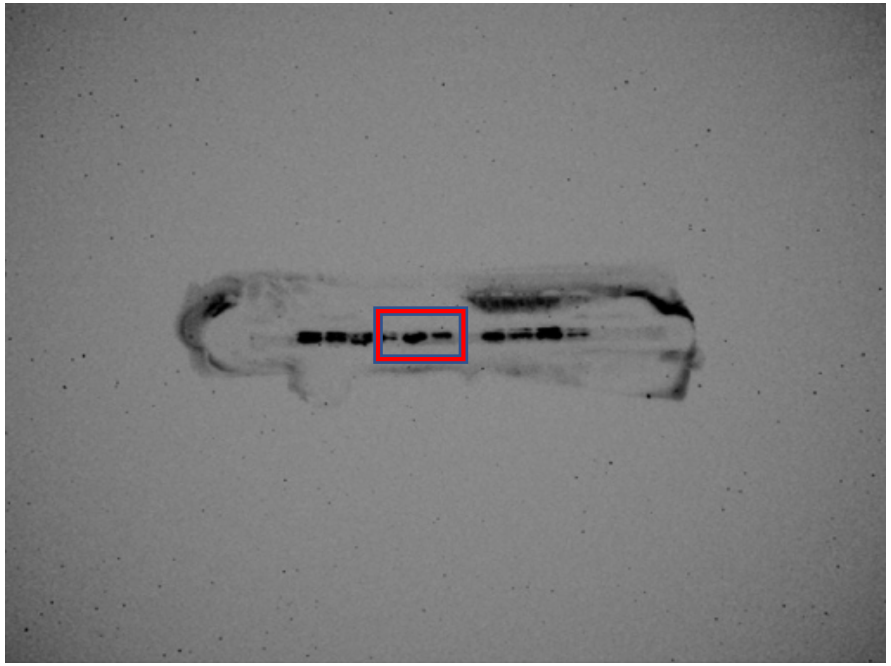

P62

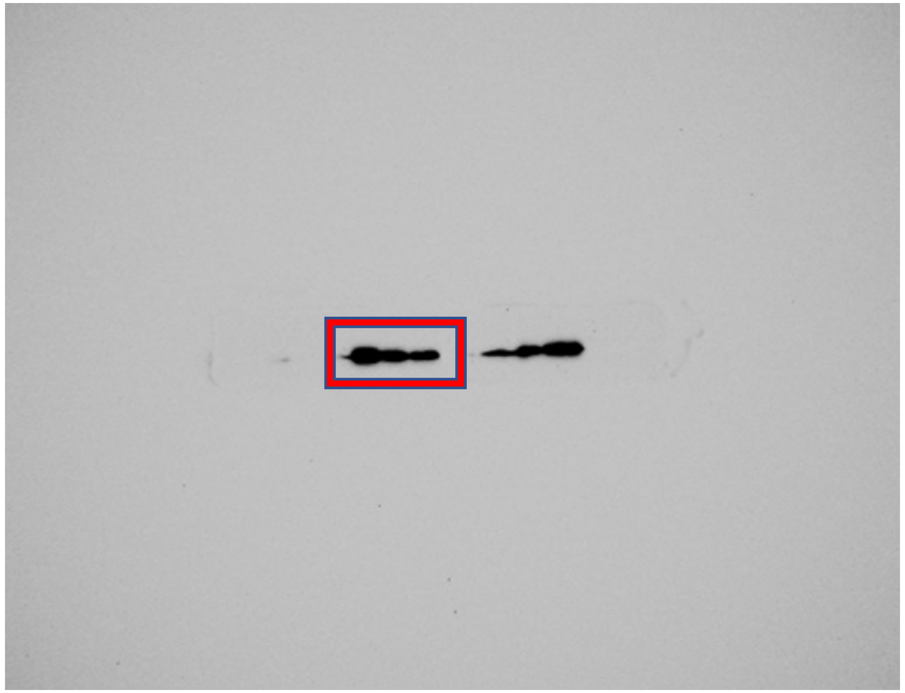

LC3B

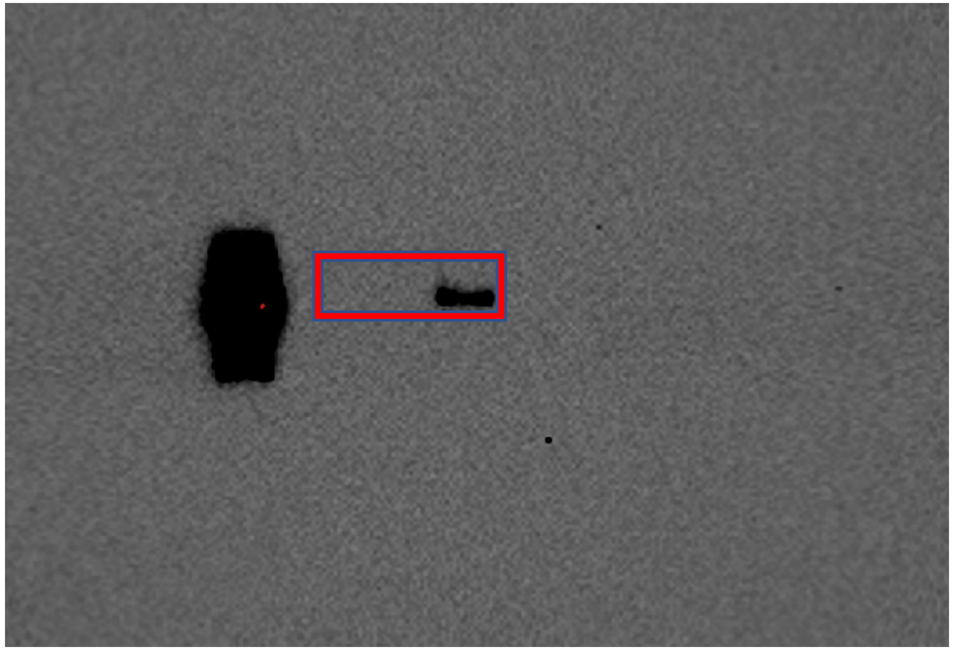

MLCK1

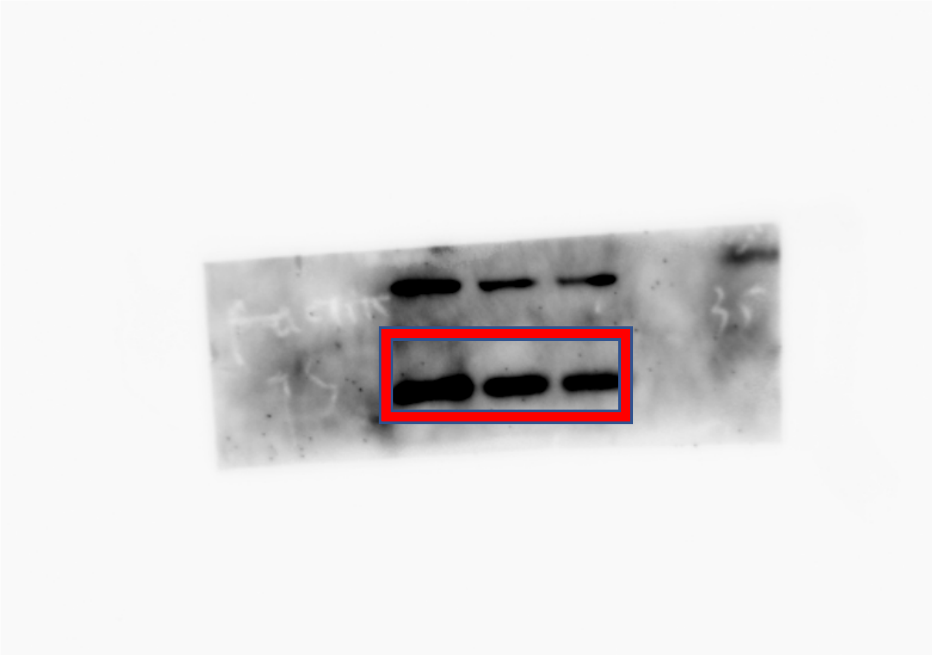

Figure4 β-actin

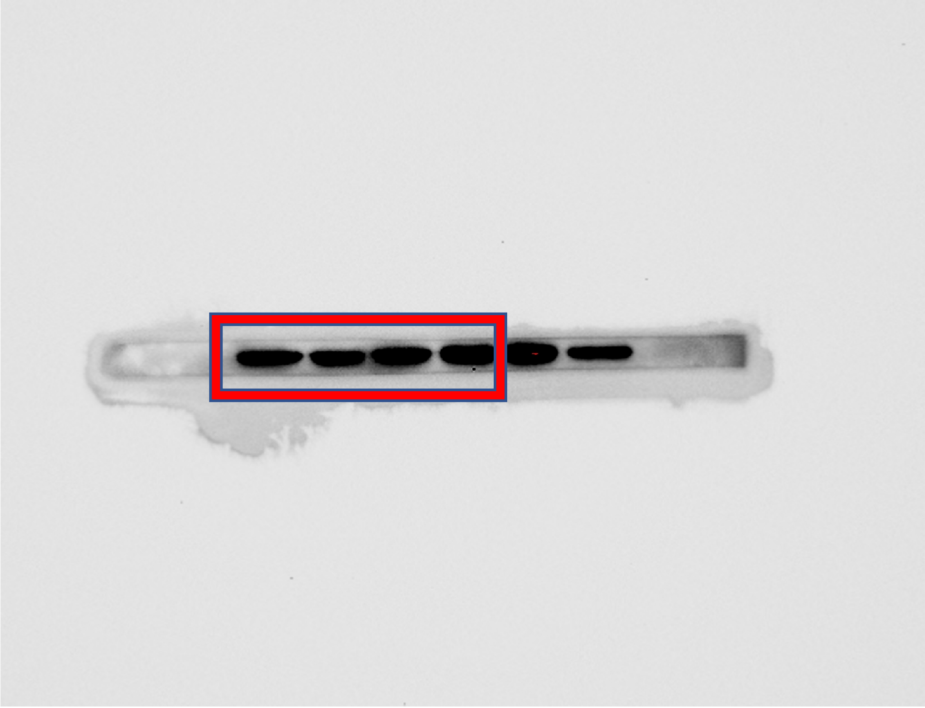

Figure3 β-actin
